# The Timed Up and Go test predicts frailty in patients with COPD

**DOI:** 10.1038/s41533-022-00287-7

**Published:** 2022-07-06

**Authors:** Ali M. Albarrati, Nichola S. Gale, Margaret M. Munnery, Natasha Reid, John R. Cockcroft, Dennis J. Shale

**Affiliations:** 1 School of Healthcare Sciences, Cardiff University, University Hospital of Wales, Cardiff, CF14 4XN UK; 2 School of Health Sciences, Cardiff Metropolitan University, Llandaff Campus, Western Avenue, Cardiff, CF5 2YB UK; 3 Centre for Health Services Research, Faculty of Medicine, Princess Alexandra Hospital, The University of Queensland, St Lucia, Australia; 4 Present Address: College of Applied Medical Sciences, King Saud University, Riyadh, Saudi Arabia

**Keywords:** Chronic obstructive pulmonary disease, Diagnosis

## Abstract

The Timed Up and Go (TUG) is a global measure of mobility and has the ability to detect frail individuals. Frail patients with chronic obstructive pulmonary disease (COPD) are usually undiagnosed. We hypothesised that the TUG would identify frail patients with COPD. Frailty was assessed in 520 patients diagnosed with COPD and 150 controls using a Comprehensive Geriatric Assessment questionnaire and frailty index (FI) was derived. The TUG was used to assess physical mobility. All participants were assessed for lung function and body composition. A ROC curve was used to identify how well TUG discriminates between frail and non-frail patients with COPD. The patients with COPD and controls were similar in age, sex and BMI but the patients with COPD were more frail, mean ± SD FI 0.16 ± 0.08 than controls 0.05 ± 0.03, *P* < 0.001. Frail patients with COPD had a greater TUG time (11.55 ± 4.03 s) compared to non-frail patients (9.2 ± 1.6 sec), after controlling for age and lung function (*F* = 15.94, *P* < 0.001), and both were greater than the controls (8.3 ± 1.2 sec), *P* < 0.001. The TUG discriminated between frail and non-frail patients with COPD with an area under the curve of 72 (95% CI: 67–76), and a diagnostic odds ratio of 2.67 (95% CI:1.5–4.6), *P* < 0.001. The TUG showed the ability to discriminate between frail and non-frail patients with COPD, independent of age and severity of the airflow obstruction. The TUG is a simple, easy and quick measure that could be easily applied in restricted settings to screen for frailty in COPD.

## Introduction

Patients with chronic obstructive pulmonary disease (COPD) are more prone to be frail compared to non-COPD individuals and it is not necessarily driven by age^1–4^. Frailty reflects a lack of resilience of physiological systems to abnormal stressors which can be characterised by a number of deficits including loss of musculoskeletal mass and strength, increased number of comorbidities, cognition deficit, and progressive deterioration in physical capacity^1,2,5^.

Previous studies have shown that patients with COPD have an increased risk of frailty compared with non-COPD individuals, and it is associated with increased fat mass which is directly linked with physical inactivity and comorbidities^1,2,6,7^. Increased fat mass and frailty in patients with COPD have also been linked to musculoskeletal dysfunction and physical incapacity, which are features of COPD^1,2,8,9^.

Frailty provides an overview of impairments and serves as a prognosticator for increased morbidity and mortality in patients with COPD^3,6,7^. A number of studies found that most patients with COPD were frail and had limited physical capacity^2,6,10–13^. However, neither frailty nor physical capacity is routinely assessed in clinical practice for several reasons, including lack of time, inappropriate space, or the requirement for the completion of lengthy questionnaires^1,6,7^. Rapid identification of a frail patient with COPD would allow early medical and rehabilitation interventions, which have been shown to reverse frailty in patients with COPD^2^.

The Timed Up and Go (TUG) test is an integrated assessment tool for lower extremity muscles strength, gait speed, balance and cognition^14^. We have previously shown that it is a simple, reliable, and valid tool for assessing physical capacity in patients with COPD, and has the ability to discriminate between physically active and inactive patients with COPD^8^. TUG captures various age-related physiological changes and discriminates between frail and non-frail older individuals^15^. As TUG is a rapid standardised test of physical capacity and overcomes limitations associated with other assessment tools of frailty, this makes it a useful proxy measure of frailty in COPD^15,16^. Therefore, this study aimed to assess the ability of the TUG to discriminate between frail and non-frail patients with COPD, and the potential to predict frailty in the patients with COPD.

## Methods

### Study design and participants

This was a cross-sectional study in community-based patients with COPD, confirmed by post-bronchodilator spirometry at enrolment. Patients with COPD were drawn from the prospective Assessment of Risk in Chronic Airways Disease Evaluation (ARCADE) study^17^. Using the G power software, a retrospective sample size calculation was based on the longitudinal ARCADE study to detect a 25% difference in the rate of frailty progression with 95% power at *P* = 0.05 level, the estimated sample size was 522.

All the patients with COPD were clinically stable, not having taken antibiotics or oral corticosteroids in the previous 4 weeks prior to recruitment. The patients with COPD with inflammatory diseases such as rheumatoid arthritis, oral maintenance corticosteroids, inflammatory bowel syndrome, and long-term oxygen therapy were excluded. The patients with COPD were recruited from respiratory outpatient clinics, pulmonary rehabilitation and smoking cessation referrals and general practice databases. We aimed to recruit patients with COPD with mild, moderate, and severe disease who had more variable clinical presentations, therefore more patients with COPD were recruited than the controls. Volunteer controls free from respiratory disease and other exclusion criteria applied to the patients with COPD were recruited to explore if the relationship of TUG with frailty remained beyond the impact of respiratory impairment. Volunteer controls were recruited from smoking cessation clinics, the patients’ relatives, and previous respiratory research databases at Cardiff University. All controls who met the inclusion and exclusion criteria were contacted before their appointment and sent a questionnaire to complete about their medical, physical, occupational and psychosocial status. All participants gave written informed consent and the study had approval from the South East Wales Research Ethics Committee (Clinical Trials.gov, NCT01656421).

### Frailty

A modified version of the comprehensive geriatric assessment (CGA) questionnaire^1,18^, specific to community-dwelling individuals was administered by a researcher to all participants, and a frailty index (FI) was derived. The FI is a reliable and valid tool for quantifying health status, stratifying patients’ risk of institutionalisation and death^18^. The FI was calculated by dividing the total number of CGA deficits by the maximum possible score of 61. The upper 90th centile FI for controls (0.09) was used as a cutoff for frailty based on a previous study by Gale and coauthors^1^.

### Lung function

All participants performed spirometry (Vitalograph Alpha, using the global lung initiative (GLI) reference equations, to determine forced expiratory volume in one second (FEV_1_), forced vital capacity (FVC) and the FEV_1_:FVC ratio^19^. A diagnosis of COPD was confirmed as post-bronchodilator spirometry FEV_1_:FVC < 0.70^20^. The patients with COPD were classified according to the Global Initiative of Obstructive Lung Disease (GOLD) combined assessment, GOLD A-D based on the COPD assessment test (CAT) score^20^.

### Body composition

Body compositions were measured barefoot in lightweight indoor clothing. Fat percentage, fat-free mass^21^ and body mass index (BMI kg/m^2^) were determined using a segmental bio-electrical impedance analyser (BC418 Tanita Corp, Tokyo). Waist circumference was measured using stretch-resistant tape.

### Timed Up and Go

All participants undertook the TUG test using a standard chair (seat height 45 cm high) and standardised instructions^22^. Participants were seated with their backs supported against the chair. They were instructed to stand up, walk three metres to a mark on the floor, cross the mark, turn around, walk back to the chair and sit down. The task was performed at their normal comfortable pace. A stopwatch was started on the word “go” and stopped as the subject sat down and the time recorded in seconds. The TUG of 8.42 sec has been previously a validated cutoff value for normal physical capacity in patients with COPD^8^.

### Handgrip measurement

Maximal right and left handgrip strength (HGS) was determined twice and the mean was calculated for each hand using a hand dynamometer (T.K.K. 5401 grip-D, Takei, Japan).

### COPD health status questionnaire

The patients with COPD completed the St George’s Respiratory Questionnaire (SGRQ) and the CAT, both validated to assess the impact of COPD on health status^23,24^.

The patients with COPD reported the number of chest exacerbations (defined as worsening of respiratory symptoms requiring antibiotic or oral corticosteroid therapy) per year^20^. Breathlessness score was recorded using the modified Medical Research Council (mMRC). The number of previously diagnosed comorbidities in the patients with COPD and controls were also recorded.

### Statistical analysis

Data analysis was performed using IBM SPSS Statistics for Windows, Version 27.0 (IBM Corp., Armonk, NY, USA). Interval ratio data were checked for normality prior to analysis. Data that were normally distributed were presented as mean and standard deviation (SD) or median (range) for non-normal and percentage for categorical data. Comparisons between the patients with COPD and controls were performed using analysis of variance. Categorical data were analysed using the Chi-square test. Relationships between variables were explored using Pearson’s and Spearman correlation coefficients. The receiver operating characteristics (ROC) curve was performed to determine the diagnostic ability of the TUG test for discrimination between frail and non-frail patients with COPD. For all analyses a *P* value < 0.05 was considered significant. The 90th percentile of FI in the controls was used as a cutoff for frailty based on a previous study, which used the same tool to detect frailty in the patients with COPD^1^. The cutoff for frailty was 10 sec as reported in a recent systematic review^25^.

### Reporting summary

Further information on research design is available in the Nature Research Reporting Summary linked to this article.

## Results

### Participant characteristics

**Fig. 1:**
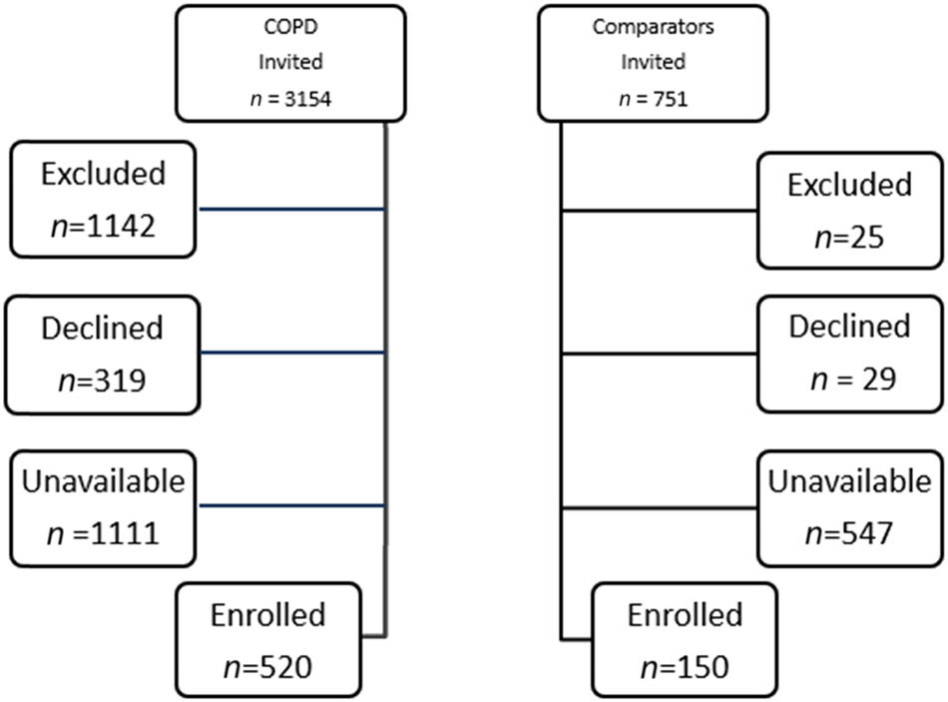
A flow chart describes the process of recruitment and enrollment of patients with COPD and controls. A flow chart describes the process of recruitment.

The process of recruitment and enrollment for this study is described in Fig. 1. The patients with COPD and controls were similar in age, sex and BMI. The patients with COPD had greater frailty index, 0.16 ± 0.08 than the controls, 0.05 ± 0.03, *P* < 0.001. The patients with COPD also had lower FEV_1_, FVC and FEV_1_/FVC, greater smoking history, larger waist circumference (All *P* < 0.001) (Table 1). The patients with COPD had greater number of diagnosed comorbidities, median (IQR) 2 (1–3) than 1(0–3) controls, *P* < 0.001.

**Table 1:** Participants physical and clinical characteristics.

	COPD (*n* = 520)	Control (*n* = 150)	*P*
Gender male: female	270:250	76:74	0.451
Age (years)	66.1 ± 7.6	65 ± 7.4	0.109
FEV_1_/FVC (%)	0.53 ± 0.11	0.78 ± 0.05	0.001
FEV_1_ (% predicted)	58 ± 19	105 ± 14	0.001
FVC (% predicted)	87 ± 21	109 ± 15	0.001
Smoking (pack years)	41 ± 25	22 ± 18	0.001
BMI (kg/m^2^)	28.0 ± 5.5	28.1 ± 4.1	0.951
Waist circumference (cm)	99.6 ± 15.0	94.7 ± 10.2	0.001
Handgrip (kg)	27.1 ± 9.7	31.3 ± 10.3	0.001
TUG (sec)	11.5 ± 4	8.3 ± 1.2	0.001
CGA total	9 (6–13)	2.25 (1–4)	0.001
Frailty index	0.16 ± 0.08	0.05 ± 0.03	0.001
mMRC	2.0 ± 1	-	-
SGRQ Total	53 (36–68)	-	-
CAT score	21 (14–27)	-	-

All data mean ± SD or Median (IQR), #=Geometric mean, *-* not assessed.

*P* < 0.05 significant difference between patients with COPD and controls

*BMI* body mass index; *CAT* COPD assessment test; *CGA* comprehensive geriatric assessment; *FEV*_*1*_ forced expiratory volume in 1 sec; *FFMI* fat-free mass index; *FVC* forced vital capacity; *mMRC* modified Medical Research Council; *SGRQ* St George’s Respiratory Questionnaire; *TUG* Timed Up and Go.

### Relationship between frailty and Timed Up and Go test, patients with COPD characteristics

In patients with COPD, both the FI and TUG were related to BMI (*r* = 0.12, *r* = 0.24), fat mass (*r* = 0.13, *r* = 0.21), waist (*r* = 0.24, *r* = 0.22), handgrip (*r* = −0.26, *r* = 0.27), number of exacerbations (*rs* = 0.14, *rs* = 0.24), SGRQ (*r* = 0.48, *r* = 0.37) and CAT (*r* = 0.45, *r* = 0.37), respectively, all (*P* < 0.05).

In patients with COPD, 210 were infrequent exacerbators, 0–1/year, while 310 were frequent exacerbators, >2/year. The FI and TUG were greater in frequent exacerbators, FI = 0.19 ± 0.09 and 11.5 ± 3.5 sec compared to infrequent exacerbators, 0.15 ± 0.08 and 10.6 ± 3.9 sec, respectively, all *P* < 0.001. Both had a greater FI than that of the control group, 0.05 ± 0.04 (*P* < 0.001).

The FI was related to the TUG in the patients with COPD, *r* = 0.50, *P* < 0.001, but not in the controls, *P* > 0.05. Based on the upper 90th centile of FI for controls (0.09) as a cut-off for frailty, 76% of the patients with COPD were frail compared to 13% of controls who were frail. Frail patients with COPD had a greater TUG time (11.55 ± 4.03 sec) compared to non-frail patients with COPD (9.2 ± 1.6 sec), *P* < 0.001, even after controlling for age and lung severity (F = 15.94, *P* < 0.001). Both groups had a greater TUG time than the controls (8.3 ± 1.2 sec), *P* < 0.001. Frail patients with COPD were younger, had lower % FEV_1_ and handgrip, and greater BMI, waist circumference and fat percentage compared to non-frail patients with COPD, all *P* < 0.05.

### Diagnostic ability of TUG test against frailty

**Fig. 2:**
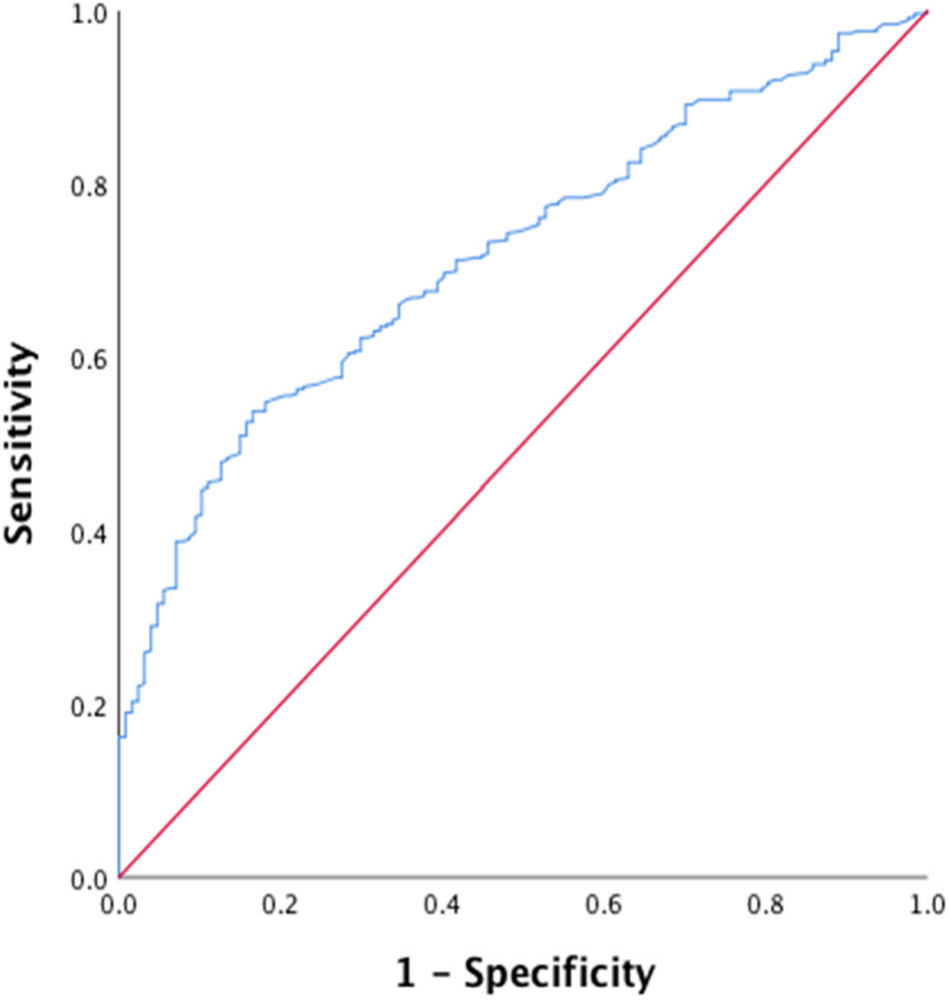
The ROC curve for the TUG test. Diagnostic ability of TUG test against frailty in the patients with COPD. Frailty is defined as frailty index ≥0.09.

Using the upper 95%CI of TUG for controls (8.42 sec) as a normal physical capacity test, 82% of the patients with COPD had impaired physical capacity. This cut-off value of TUG test identified 85% of frail patients with COPD based on the upper 90^th^ centile FI for controls (0.09) as a cut-off for frailty. Using this cut-off for frailty to determine the best cut-off value for the TUG, with 90% sensitivity and 80% specificity, the FI cut-off value of 0.09 corresponds to 8 sec on the TUG with 90% positive predictive value. The diagnostic odd ratio of 2.67 (95%CI:1.5–4.6) area under the curve was 72 (95%CI: 67–76), *P* < 0.001 (Fig. 2). Using the cut-off of 10 sec for frailty as reported in recent systematic review was found to have 86% sensitivity and 62% specificity with a positive predictive value of 64% and diagnostic odd ratio of 3.6 (95%CI:2.4–5.6), and the area under the curve (UAC) was 76 (95%CI: 72–80).

## Discussion

To our knowledge, this is the first study to examine the TUG against the validated CGA tool in elderly or diseased population. In this study, frail patients with COPD took longer time to complete a simple physical capacity test compared to non-frail patients with COPD. The TUG test demonstrated the ability to discriminate between frail and non-frail patients with COPD.

The TUG is a complex test that requires multiple integrated and coordinated tasks that involve sufficient muscles strength, gait speed, balance and substantial cognition^25^. The TUG test reflects physical capacity in patients with COPD and elderly, and captures several physiological and physical features that are not specifically age related^8,25–27^. Previous studies in community elderly individuals showed that frail elderly took longer time to complete the TUG test compared to non-frail elderly^28–30^. Frailty and COPD share similar physiological and physical features where both are characterised by reduced physical capacity. Reduced physical capacity is multifactorial in patients with COPD resembling frailty. The TUG and FI were associated with both clinical and physical manifestations of COPD. Interwoven relationships between frailty and physical capacity in COPD look similar to what is observed in the natural aging process and accumulation of deficits such as sarcopenia, increased fat tissues and inflammation. Previous studies on the elderly and COPD have shown that frailty could be predicted by physical capacity^1,2,7,10,12,28,29,31^. The lack of association between TUG and frailty in controls may be because only 13% of controls were frail, based on the upper 90^th^ centile FI for controls (0.09) as a cut-off for frailty. Other studies, in contrast, have reported an association between TUG and frailty in their control group. These studies have included individuals with chronic diseases such as cardiorespiratory diseases, type 2 diabetes mellitus and chronic kidney disease, which are well-established risk factors for physical impairment and increased risk of frailty^28,29,31^.

In our study, we showed that the TUG test has the ability to identify frail patients with COPD, as well as to predict frailty. The TUG time of 8 sec in our study identified 90% of frail patients with COPD, and is similar to a previous study in Brazilian elderly population^31^. Likewise, previous studies examined the TUG against other frailty tools and demonstrated that the TUG time above 8 sec is sensitive to detect frailty elements in elderly individuals^15,26,29,30,32,33^. Several studies showed that the subtasks of the TUG test are as good and sensitive as other traditional measures, including gait speed, in discriminating frail individuals^15,16,25,31^. Filippin and colleagues found that the TUG time could discriminate between frail and non-frail elderly individuals using the Fried Frailty Criteria independently of age^31^. Using a cut-off of 10 sec showed similar sensitivity results to 8 sec to detect frail patients with COPD, however, it had lower specificity. This is similar to what has been reported in the recent systematic review, which found that a cutoff of 10 sec is highly sensitivity, 93%, but not very specific with 38% of elderly individuals being incorrectly classified as frail^25^.

Physiological deficits occur with aging, including loss of skeletal muscle mass and strength with increased loss of physical activity, increased number of comorbidities, resembles the impact of multiple comorbidities seen in COPD^34^. This accumulation of physiological and physical deficits in COPD resembles the pattern that occurs with aging healthy populations, which are associated with increased risk of frailty^1,4,10,12,35^. In our study, we found both the TUG test and frailty are associated with the most common features seen in COPD such as peripheral musculoskeletal weakness, body composition and increased number of comorbidities. Thus, the TUG test is likely to capture similar manifestations that are associated with frailty, independent of the severity of airflow obstruction^8,36^. This is consistent with previous studies, which found that the TUG test is a measure of the interaction of body composition, muscle strength and comorbidities on the physical mobility and frailty in elderly^15,16,26,29,31,32^.

Integrating an objective measure such as the TUG, which demonstrated the ability to capture frailty features in COPD and detected the patients with COPD with more breathlessness, poor symptoms and health status would help clinicians in tailoring appropriate personlised management^29–32^. Additionally, using validated simple and quick self-administered questionnaires with a physical activity measure would be suitable for tele-medicine and tele-rehab clinics to monitor a patient’s health status.

This study has several limitations, including its cross-sectional nature, which means the findings may be influenced by bias, confounding variables and lack of causality. Although the patients with COPD and controls were similar in age and BMI, they differed in smoking history and comorbidities, all of which may influence the results. In this study, we included controls with hypertension and hypercholesterolaemia as it is difficult to find healthy smoker controls. Additionally, we did not exclude the patients with COPD with overt cardiovascular disease, but we minimised the risk of confounding factors by excluding other inflammatory diseases such as cancer and inflammatory bowel disease. The CGA questionnaire assesses frailty based on a patient’s recall, however many of the CGA’s items were verified by medical notes, clinical examination and carers.

The TUG showed the ability to discriminate between frail and non-frail patients with COPD, independent of age and severity of the airflow obstruction. The TUG is a simple, easy and quick measure that provides a global assessment of physical mobility in COPD. It is a standardised, integrated test that captures multisystem deficits, which could be easily applied by clinicians in restricted settings as a screening tool for frailty in patients with COPD.

## Supplementary information

REPORTING SUMMARY

## Data Availability

The data sets generated during and/or analyzed during the current study are available from the corresponding author on reasonable request.
