# Supplementary material for: The Timed Up and Go test predicts frailty in patients with COPD
Source: NPJ Prim Care Respir Med. 2022 Jul 6;32:24. doi: 10.1038/s41533-022-00287-7 (PMC9259691; doi:10.1038/s41533-022-00287-7)
Supplement: Supplementary file 1 — REPORTING SUMMARY [file 41533_2022_287_MOESM1_ESM.pdf]

nature portfolio

Corresponding author(s): Ali Albarrati
Rehabilitation Sciences Department,
College of Applied Medical Sciences,
King Saud University,
P.O Box 10219
Riyadh 11433
KSA.
Email: albarrati@ksu.edu.sa
Tel/Fax: +966114696128

Last updated by author(s): Jul 13, 2021

Reporting Summary

Nature Portfolio wishes to improve the reproducibility of the work that we publish. This form provides structure for consistency and transparency in reporting. For further information on Nature Portfolio policies, see our Editorial Policies and the Editorial Policy Checklist.

Statistics

For all statistical analyses, confirm that the following items are present in the figure legend, table legend, main text, or Methods section.

n/a	Confirmed	
☐	☒	The exact sample size (n) for each experimental group/condition, given as a discrete number and unit of measurement
☐	☒	A statement on whether measurements were taken from distinct samples or whether the same sample was measured repeatedly
☐	☒	The statistical test(s) used AND whether they are one- or two-sided<br>*Only common tests should be described solely by name; describe more complex techniques in the Methods section.*
☐	☒	A description of all covariates tested
☐	☒	A description of any assumptions or corrections, such as tests of normality and adjustment for multiple comparisons
☐	☒	A full description of the statistical parameters including central tendency (e.g. means) or other basic estimates (e.g. regression coefficient) AND variation (e.g. standard deviation) or associated estimates of uncertainty (e.g. confidence intervals)
☐	☒	For null hypothesis testing, the test statistic (e.g. F, t, r) with confidence intervals, effect sizes, degrees of freedom and P value noted<br>*Give P values as exact values whenever suitable.*
☒	☐	For Bayesian analysis, information on the choice of priors and Markov chain Monte Carlo settings
☐	☒	For hierarchical and complex designs, identification of the appropriate level for tests and full reporting of outcomes
☐	☒	Estimates of effect sizes (e.g. Cohen's d, Pearson's r), indicating how they were calculated

Our web collection on statistics for biologists contains articles on many of the points above.

Software and code

Policy information about availability of computer code

Data collection	NA
Data analysis	NA

For manuscripts utilizing custom algorithms or software that are central to the research but not yet described in published literature, software must be made available to editors and reviewers. We strongly encourage code deposition in a community repository (e.g. GitHub). See the Nature Portfolio guidelines for submitting code & software for further information.

Data

Policy information about availability of data

All manuscripts must include a data availability statement. This statement should provide the following information, where applicable:

- Accession codes, unique identifiers, or web links for publicly available datasets
- A description of any restrictions on data availability
- For clinical datasets or third party data, please ensure that the statement adheres to our policy

Data are available on request due to privacy or other restrictions

Field-specific reporting

Please select the one below that is the best fit for your research. If you are not sure, read the appropriate sections before making your selection.

☒ Life sciences ☐ Behavioural & social sciences ☐ Ecological, evolutionary & environmental sciences

For a reference copy of the document with all sections, see nature.com/documents/nr-reporting-summary-flat.pdf

Life sciences study design

All studies must disclose on these points even when the disclosure is negative.

Sample size	Using the G power software, the sample size was calculated based on the longitudinal ARCADE study to detect a 25% difference in the rate of progression with 95% power at p = 0.05 level, the estimated sample size was 522 would be required.
Data exclusions	Patients with COPD with inflammatory disease such as rheumatoid arthritis, oral maintenance corticosteroids, inflammatory bowel syndrome and long-term oxygen therapy were excluded.
Replication	Frailty, lung function, body composition, hand-grip strength, Timed up and Go test, SGRQ, CAT
Randomization	NA
Blinding	NA

Reporting for specific materials, systems and methods

We require information from authors about some types of materials, experimental systems and methods used in many studies. Here, indicate whether each material, system or method listed is relevant to your study. If you are not sure if a list item applies to your research, read the appropriate section before selecting a response.

Materials & experimental systems

n/a	Involved in the study	
☒	☐	Antibodies
☒	☐	Eukaryotic cell lines
☒	☐	Palaeontology and archaeology
☒	☐	Animals and other organisms
☐	☒	Human research participants
☒	☐	Clinical data
☒	☐	Dual use research of concern

Methods

n/a	Involved in the study	
☒	☐	ChIP-seq
☒	☐	Flow cytometry
☒	☐	MRI-based neuroimaging

Human research participants

Policy information about studies involving human research participants

Population characteristics	patients with COPD were clinically stable, not having taken antibiotics or oral corticosteroids in the previous 4 weeks prior to recruitment
Recruitment	Patients with COPD were recruited from respiratory outpatient clinics, pulmonary rehabilitation and smoking cessation referrals, general practice databases. Controls free from respiratory disease and other exclusion criteria applied to the patients with COPD were recruited to explore if the relationship of TUG with frailty remained beyond the impact of respiratory impairment. Controls were recruited from smoking cessation clinics, patents' relatives and previous respiratory research databases at Cardiff University. All participants were contacted before their appointment and sent a questionnaire to complete about their medical, physical, occupational and psychosocial status.
Ethics oversight	The study had approval from the South East Wales Research Ethics Committee.

Note that full information on the approval of the study protocol must also be provided in the manuscript.
